# The dissemination of ST80-SCC*mec*-IV community-associated methicillin resistant *Staphylococcus aureus *clone in Kuwait hospitals

**DOI:** 10.1186/1476-0711-9-31

**Published:** 2010-11-04

**Authors:** Edet E Udo, Eiman Sarkhoo

**Affiliations:** 1 Department of Microbiology, Faculty of Medicine, Kuwait University, Kuwait

## Abstract

**Background:**

Community-associated methicillin-resistant *Staphylococcus aureus *(CA-MRSA) is a global healthcare problem. The purpose of this study was to characterize CA-MRSA clones and their distribution in Kuwait hospitals.

**Methods:**

In total, 135 CA-MRSA isolates, carrying the SCCmec IV or V genetic elements, isolated in eight hospitals were characterized using antibiogram, pulsed-field gel electrophoresis, multilocus sequence typing, and carriage of genes for Panton-Valentine Leukocidin (PVL), capsular polysaccharides types (cap) 5 and 8, accessory genes regulators (agr), Staphylococcal enterotoxins (SE) and toxic shock syndrome toxin 1 (*tst*).

**Results:**

They were susceptible to vancomycin, teicoplanin and linezolid but resistant to kanamycin (62%), fusidic acid (42.2%), tetracycline (39.3%), erythromycin and clindamycin (21.5%), gentamicin (5.9%), streptomycin (6.7%), trimethoprim (5.9%), mupirocin (6.6%) and cadmium acetate (82.2%). They consisted of 10 pulsotypes with the majority belonging to PFGE type I (51.1%), type II (22.2%), type IV (13.3%) and type III (3.7%). They belonged to 10 sequence types (ST) comprising ST80 (51.1%), ST30 (22.2%), ST5 (14.1%), ST1 (4.45), ST6 (3.7%), ST88 (1.5%), ST834 (1.5%), ST8 (0.7%), ST46 (0.7%) and ST950 (0.7%). Genes for PVL, cap 8, cap 5 and agr III, agr I and agr II were detected in 61.5%, 77.3%, 20.7% and 62.2%, 17% and 8.1% of the isolates respectively. Nine (6.7%) isolates contained *tst *while 103 isolates were positive for SE genes with *sei *(63.0%), *seg *(41.5%) and *sed *(29.6%) as the common SE genes.

**Conclusions:**

ST80-SCC*mec*IV was the most common CA-MRSA clone in Kuwait hospitals presenting new challenges for infection control.

## Background

Following their initial reports from patients in remote communities with no access to healthcare facilities [1-3], Community-associated methicillin resistant *S. aureus (*CA-MRSA) has now been reported in many countries and has become a global public health problem [4,5]. CA-MRSA carry the smaller staphylococcal chromosome cassette mec (SCC*mec*) types IV and V whereas the larger SCC*mec *types I, II and III are present in MRSA traditionally associated with infections in healthcare facilities [4,5]. Initially, CA-MRSA were generally more susceptible to non beta-lactam antibiotics than healthcare associated MRSA [1,4,5]. However, some CA-MRSA strains have now acquired multidrug resistance [6-9].

The transmission of CA-MRSA was restricted to particular populations with distinctive risk factors such as injections drug users and aboriginal communities [2,4,5]. However, since the 1990's CA-MRSA has become widespread and have been introduced from the community into healthcare facilities where, in some instances, they have become the dominant MRSA clones [10,11]. They have demonstrated fitness to survive in healthcare facilities. Consequently, some epidemic CA-MRSA clones have emerged in several locations. These include the USA300 clone which is dominant in USA and North America [12], the ST30 (Oceanic clone) [13] and the European ST80 clone [14-16].

CA-MRSA was first isolated in small numbers in Kuwait hospitals in 2001 [17] when it constituted 1.8% of MRSA obtained from patients in seven hospitals. Molecular typing of these isolates revealed ST30 and ST80 as the common CA-MRSA clones [17]. Since then, the prevalence of CA-MRSA has increased substantially and by 2005, 17% of all MRSA isolated in Kuwait hospitals were CA-MRSA [18]. However, there was no information on the clonal composition of these strains. The objective of this study was to characterize CA-MRSA obtained in Kuwait hospitals between 2005 and 2006 using SCC*mec *typing, PFGE and multi-locus sequence typing to ascertain their clonal composition and prevalence.

## Materials and methods

### MRSA isolates

From January 2005 to December 2006, the MRSA Reference Laboratory, located at the Department of Microbiology, Faculty of Medicine, Kuwait University, Kuwait, received 889 MRSA for typing. One hundred and thirty five of these isolates were classified as CA-MRSA based on their carriage of SCC*mec *IV or V genetic elements and urease production. The 135 isolates originated from 131 patients located in eight hospitals. The isolates were collected from Mubarak (MBH, 18 isolates), Amiri (AMH,31 isolates), Al-Sabah (ASH,29 isolates), Farwania (FAH; 12 isolates), Ibn Sina (ISH; 16 isolates), Al-Razi (ARH; 9 isolates), Jahra (JH; 19 isolates) and the Maternity hospital (MAT;1 isolate). from wound swabs (37 isolates), abscesses (18 isolates), nasal swabs (18 isolates), ear swabs (9 isolates), blood samples (5 isolates), groin (5 isolates), throat swabs (5 isolates), urine samples (4 isolates), skin swabs (3 isolates), tracheal aspirates (3 isolates), high vaginal swabs (2 isolates), sputum (2 isolates) and eye swab (1 isolate). The source for 20 isolates was not provided. The four isolates from the same patient were obtained from four different sites and were all resistant to mupirocin [9]. Isolates were identified at the diagnostic laboratories and confirmed as CA-MRSA at the MRSA Reference Laboratory using SCC*mec *typing and urease production. Pure cultures were preserved in 40% (v/v) glycerol in brain heart infusion broth (BHIB) at -20°C. Isolates were recovered in BHIB, incubated at 37 °C, and sub-cultured on brain heart infusion agar plates before testing.

### Susceptibility to antibacterial agents

Susceptibility testing to antibacterial agents was performed by the disk diffusion method [19] using oxacillin, benzyl penicillin, cefoxitin, kanamycin, gentamicin, erythromycin, clindamycin, chloramphenicol, tetracycline, trimethoprim, fusidic acid, rifampicin, ciprofloxacin, and linezolid. Susceptibility to fusidic acid was interpreted following guidelines by the British Society for Antimicrobial Chemotherapy [20]. Disks containing (values per disk): cadmium acetate (50 μg), propamidine isethionate (100 μg), mercuric chloride (109 μg) were prepared in the laboratory. The results were interpreted as reported previously [20]. Disk susceptibility testing for mupirocin was performed with disks containing 5 μg and 200 μg of mupirocin (Oxoid, Basingstoke, UK). Growth to the edge of both the 5 μg and the 200 μg disks indicated high level resistance. Growth to edge of the 5 μg disk alone defined low level resistance [21]. High-level mupirocin resistance was confirmed by MIC determination and the detection of mupA gene by PCR as described previously [9]. The MIC for oxacillin, mupirocin, vancomycin and teicoplanin were determined with Etest strips (AB Biodisk, Solna, Sweden) according to the manufacturer's instructions. The Macro method, using an inoculum equivalent to 2× McFarland standard on brain heart infusion agar, was used to determine MIC for vancomycin and teicoplanin [22]. *S. aureus *strain ATCC25923 was used as quality control strain for susceptibility testing. Methicillin resistance was confirmed by detecting PBP 2a using a rapid latex agglutination kit (Denka-Seiken, Japan) according to the manufacturer's instructions.

### Urease production

Urease production was detected on Christensen's urea agar slope after incubation at 35°C for 48 hours.

### SCC*mec and ccr *typing

Staphylococcal cassette chromosome *mec *(SCC*mec*) typing and subtyping were performed as described previously [23]. The cassette chromosome recombinase (ccr complex) types was determined using primers and methods described previously [24]. Strain COL (SCC*mec *type 1, ccr 2); XU642 (EMRSA-16, SCC*mec *type II, ccr 2); WBG525 (EMRSA-1, SCC*mec *type III), and K1814 (SCC*mec *type IV) and WBG 8318(SCCmec type V) as controls.

### Pulsed-field gel electrophoresis (PFGE)

Genomic DNA was prepared in agarose plugs as described previously [25]. DNA was digested with 40 U *Sma *I- (Biolabs, New England, USA) for 4 hr at 25°C. Counter-clamped homogenous electric field (CHEF) electrophoresis of digested chromosomal DNA was performed in 1.2% agarose (pulse-field certified agarose, BioRad, USA) at 6 V/cm for 21 h at 14°C, with pulse times of 5 s-40 s using a CHEF-DRIII system (BioRad). *Sma *I digested chromosomal DNA of *S. aureus *strain NCTC8325 was used as a molecular size marker. After electrophoresis, the gel was stained with ethidium bromide 0.5 μg/ml and photographed under ultraviolet illumination. The chromosomal patterns were examined visually and similarities of the PFGE patterns were assigned using criteria published previously [26].

### Multilocus sequence typing (MLST)

Multilocus sequence typing (MLST) was performed by following previously published protocols [27]. The sequences of the seven house keeping genes (*arc, aroE, glpF, gmk*, *pta, tpi *and *yqiL*) were compared to existing sequences in the MLST database http://www.mlst.net for assignment of allelic numbers. Sequence types (ST) were assigned according to their allelic profiles.

### Detection of virulence genes

DNA was extracted from *S. aureus *for PCR following a protocol described previously [25]. PCR assays were used to investigate the carriage of genes for accessory gene regulator (agr) types I, II, III and IV using the primers and conditions published previously [28]. The LukS-PV-lukF-PV gene which expresses Panton-Valentine leucocidin (PVL) was amplified as described previously [15]. The amplification of genes for capsular polysaccharide (cap) types 5 and 8 were performed using primers and conditions described previously [28]. Genes for the staphylococcal enterotoxins, *sea, seb*, *see, sed, sei, seh *and toxic shock syndrome gene, tst were amplified in multiplex PCR as described previously [25]. All PCR products were analysed by agarose gel electrophoresis on 1.5% agarose gels [25].

### Statistics

Differences in the distribution of CA-MRSA clones were assessed by Chi square test. A P-value of ≤ 0.05 was considered significant.

## Results

### Staphylococcal cassette chromosome *mec *(SCC*mec*) and ccr typing

The 135 MRSA isolates included in this study produced urease and belonged to SCC*mec *type IV or type V. Urease positivity was used to differentiate CA-MRSA carrying the SCC*mec *IV genetic element from the healthcare-associated epidemic MRSA-15 (EMRSA-15) which also carries the SCC*mec*-IV genotype but does not produce urease [29]. In total, 102 (75.6%) of the isolates carried SCC*mec *type IV, 11 (8.1%) carried SCC*mec *type IVa, 10 (7.4%) carried SCC*mec *type IVc and 12 (8.9%) the SCC*mec *type V genetic element. A total of 123 (91.1%) isolates consisting of the SCC*mec *type IV, SCC*mec *IVa and SCC*mec *IVc isolates belonged to *ccr *type 2 while the 12 SCC*mec *type V isolates belonged to *ccr *type 5.

### Antibacterial resistance of CA-MRSA isolates

**Figure 1:**
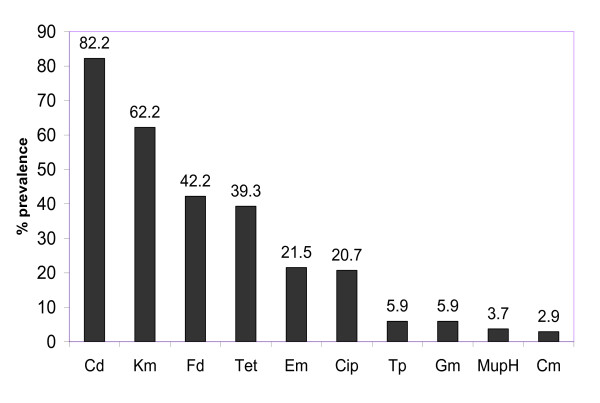
**Antimicrobial resistance of CA-MRSA isolates**. Abbreviations: Cd, cadmium acetate; Kan, kanamycin; Fd, fusidic acid; Tet, tetracycline; Erm, erythromycin; Cip, ciprofloxacin; Tp, trimethoprim; Gen, gentamicin; mupH, high-level mupirocin; Chl, chloramphenicol.

All isolates were susceptible to vancomycin MIC: ≤ 2 μg/ml), teicoplanin MIC: ≤ 2 μg/ml), rifampicin and linezolid but were resistant to oxacillin (MIC 8- > 256 μg/ml) and the antibacterial agents summarized in Figure 1. There was a high prevalence of resistance to cadmium acetate (82.2%), kanamycin (62.2%), fusidic acid (42.2%), tetracycline (39.3%), erythromycin and clindamycin (21.5%) and ciprofloxacin (20.7%). Sixty-eight (50.4%) of the 135 CA-MRSA isolates were multidrug resistant (i.e. resistant to more than three classes of non-beta lactam antibiotics tested).

### Molecular typing of CA-MRSA isolates

**Figure 2:**
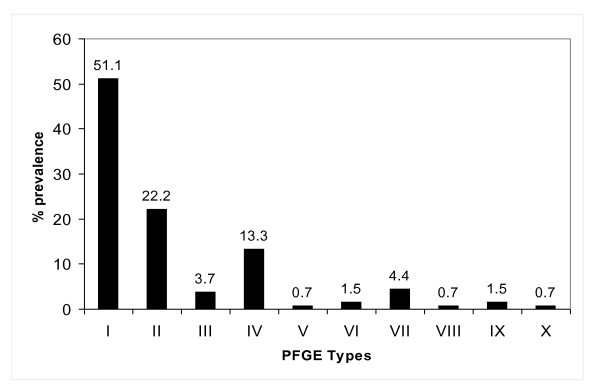
**Distribution of PFGE types among CA-MRSA isolates**.

Figure 2 shows the distribution of the 10 PFGE types detected among the 135 isolates. The majority belonged to PFGE type I or its five subtypes (51.1%), PFGE type II and its two subtypes (23.7%), PFGE type IV and subtype IVa (13.3%). The other PFGE types occurred sporadically.

MLST was performed on 28 isolates representing each of the PFGE subtypes to determine their sequence types (ST). MLST results showed extensive agreement with PFGE except for ST5 that was detected in PFGE types IV and IX. The isolate belonging to PFGE type IX was ST5 while the isolate belonging to subtype IXa was ST950 (a SLV of ST5 at *tpi*). Based on the distribution of sequence types according to their PFGE patterns, 69 (51.1%) of the 135 isolates were ST80. This was followed by ST30 (22.2%), ST5 (14.1%), ST1 (4.4%), ST6 (3.7%), ST88 (1.5%), ST834 (1.5%), ST8 (0.7%), ST46 (0.7%) and ST950 (0.7%). As shown in Table 1, the ST80 clone was present in seven of the eight hospitals. ST30 and ST5 clones were detected in six hospitals while ST1 clone was present in five hospitals. The other clones occurred sporadically. When compared with the distribution of CA-MRSA clones prevalent in Kuwait hospitals in 2001-2003 [17], the proportion of ST80 isolates increased from 38.5% in 2001-2003 [17] to 51.1% (*P *= 0.29), while the proportion of ST30 isolates decreased from 30.8% in 2001-2003 to 23.7% (*P *= 0.45)

**Table 1:** Distribution of CA-MRSA clones in Kuwait hospitals

ST	AMH	ARH	ASH	FAH	MBH	JH	ISH	MAT	Total	%
80	12	4	8	10	14	11	10	-	69	51.1
30	10	-	11	-	2	3	3	1	30	22.2
5	3	4	7	-	1	3	1	-	19	14
1	1	-	1	2	1	-	1	-	6	4.4
6	1	2	-	-	2	-	-	-	5	3.7
88	-	-	-	2	-	-	-	-	2	1.5
8	1	-	-	-	-	-	-	-	1	0.7
46	1	-	-	-	-	-	-	-	1	0.7
950	1	-	-	-	-	-	-	-	1	0.7
834	-	-	-	-	-	-	1	-	1	0.7

Total	30	10	27	14	20	17	16	1	135	

### Detection of virulence-associated genes

None of the isolates was positive for agr type IV. However, they contained genes for agr III (62.2%), agr I (17%), agr II (8.1%), capsular polysaccharides types 8 (77.3%) and type 5 (20.7%). Three (2.2%) isolates yielded negative result for both *cap 5 *and *cap 8*. The PVL gene was detected in 61.5% of the isolates.

In total, 103 (76.3%) isolates yielded positive results for *sea *(10.4%), *seb *(3.7%), *sec *(4.4%), *sed *(29.6%), *seg *(41.5%), *seh *(1.5%), *sei *(63.0%) and *tst *(6.7%). None of the isolates was positive for *see*. Twenty-seven (26.2%), 52 (50.5%), 17 (16.5%) and seven (6.8%), of the 103 isolates were positive for single, two, three and four SE genes respectively. Twenty-three isolates contained *seg *and *sei *while 20 isolates contained *sed *and *sei*.

### Diversity of ST80-CA-MRSA isolates

Table 2 summarizes the phenotypic and genotypic characteristics of the ST80 isolates. The 69 ST80 isolates had related but not identical PFGE patterns. Although 39 of the isolates had identical PFGE patterns (PFGE type I), the remaining 30 isolates were grouped into five subtypes: Ia (2 isolates), Ib (10 isolates), Ic (1 isolate), Id (2 isolates and Ie (15 isolates).

**Table 2:** Characteristics of ST80 CA-MRSA isolates.

				*Virulence-associated genes (# of positives)*		
**# of isolates**	**PFGE**	**SCC*mec***	**Antibiogram**	**PVL**	**SE**	***tst***

4	I	IV	Cd, Kan, Tet, Fd	+ (2)	*sei,(2) sed *(2)-	-
2	Ib	IV	Cd, Kan, Tet, Fd	-	*sei (1), sed (1), seg (1)*	-
1	Id	IV	Cd, Kan, Tet, Fd	+	-	-
2	Ie	IV	Cd, Kan, Tet, Fd	+	-	-
3	I	IV	Cd, Kan, Tet, Fd, Cip	+ (2)	*sei (2), sed (1)*	-
2	Ie	IV	Cd, Kan, Tet, Fd, Cip	+ (2)	*sei (2), sed (2)*	-
1	I	Iva	Cd, Kan, Tet, Fd, Cip, Gen	+	*sei, seg *,	-
4	Ib	IV	Cd, Kan, Tet, Fd, Mup	+	*seg (4)*	-
1	I	IV	Cd, Kan, Tet, Fd, Tp	+	*seg*	-
1	I	IV	Cd, Kan, Tet, Fd, Erm	+	*sei*	-
1	Ie	IV	Cd, Kan, Tet, Fd, Gen	-	*seg*	-
2	I	IV	Cd, Kan, Fd, Erm, Cip	+ (1)	*sei (1), seg (1)*	-
1	I	IV	Cd, Kan, Fd, Erm, Cip, Chl	-	*sed*	-
1	I	IV	Cd, Kan, Fd, Cip	-	*sei*	-
1	Ib	IV	Cd, Kan, Fd, Cip	+	*sei, seg*	-
1	I	IV	Cd, Kan, Fd, Gen	+	*seg*	-
1	Ib	IV	Cd, Kan, Fd, Gen	-	-	-
1	I	IV	Cd, Kan, Fd	+	*sei*	-
1	Ib	IV	Cd, Kan, Fd	+	*sei,, sed*	+
3	I	IV	Cd, Kan	+ (3)	*sei,(2) sed *(2)	-
1	Ia	IV	Cd, Kan	+	*sei*	-
1	Ie	IV	Cd, Kan, Erm, Tet	-	-	-
1	Ie	IV	Cd, Kan, Erm, Cip	-	*sei, sed*	-
2	I	IV	Cd, Kan, Erm, Cip	+ (1)	*Sei (1), seg (1)*	-
1	Ia	IV	Cd, Kan, Erm, Cip, Chl	+	-	-
1	I	IV	Cd, Kan, Cip, Tp, Gen	+	*sei*	-
7	I	IV	Cd	+	*sei (2), sed (1), seg (1)*	
1	Ie	IV	Cd	+	-	-
2	Ib	IV	Cd	-	*sei (2),, seg (2), sec(1)*	-
3	I	IV	Cd, Erm	+	*sei (3), seg (2), Sed (1)*	-
1	I	IV	Cd, Fd	+	-	-
2	I	IV	Cd, Tet	+	*Sei (1), seg(2)*	-
1	Ic	IV	Cd, Tet, Fd, Chl, Tp, Cip, Erm	-	*sei, sed, seg*	-
1	I	IV	Erm, Cip	+	*sei, seb, seg*	-
1	Ie	IVc	Kan, Tet, Fd	+	-	-
1	Ie	IV	Kan, Tet, Fd	+	*sei, sed*	-
1	Ib	IV	Kan	+	*sei, sed*	-
2	Ie	IV	Kan	+ (2)	*sei (1), sed(1)*	-
1	Ie	IV	Kan, Erm	+	-	-
1	Ie	IV	Tet	-	*sei, sed, sea*	-
1	Id	IV	-	+	*sea*	-
1	I	IV	-	+	*sei*	+
1	Ie	IV	-	-	-	-

Abbreviations:Cd, cadmium acetate; Cip, ciprofloxacin; Chl. chloramphenicol; Erm, erythromycin; Fd, fusidic acid; Gen, gentamicin; Kan, kanamycin; Tet, tetracycline; Tp, trimethoprim.

The ST80 isolates expressed low oxacillin MIC (MIC 8-64 μg/ml). They also expressed diverse resistance phenotypes. In total, 23 of the 69 isolates were resistant to kanamycin, tetracycline and fusidic acid, that are traditionally associated with ST80-SCC*mec*-IV isolates (17, 28). Of these 23 isolates, 15 expressed additional resistance to one or more of ciprofloxacin, gentamicin, erythromycin, trimethoprim, mupirocin and chloramphenicol. Furthermore, some of the isolates were susceptible to kanamycin, tetracycline and fusidic acid that usually characterize ST80-SCC*mec*-IV isolates. Ten isolates were resistant to cadmium acetate but were susceptible to all of the non beta-lactam agents tested. Forty one (59.4%) of the 69 isolates were multiresistant (resistance to more than three antibiotic classes).

All ST80 isolates carried genes for capsular polysaccharide types 8 and agr III but varied in the carriage of other virulence-associated genes. As presented in Table 2, 58 (84.0%) and 49 (71.0%) of the 69 isolates were positive for PVL and SE genes respectively. Fifteen isolates were positive for single SE gene while 30 were positive for two or more SE genes. The majority of the isolates were positive for *sei *(77.6%) and *seg *(36.7%). Nineteen (27.9%) isolates were positive for *sed *and *sei*, and ten (14.7%) were positive for *seg *and *sei*. Two isolates were positive for *tst*.

## Discussion

This study characterized ten different CA-MRSA clones in Kuwait hospitals with ST80 (51.1%) and ST30 (22.2%) as the major CA-MRSA clones similar to the composition of CA-MRSA isolates circulating in Kuwait hospitals in 2001-2003 [17]. However, results of this study differ from the previous study in three aspects. The 2001-2003 isolates consisted of five CA-MRSA clones whereas the present study has identified ten CA-MRSA clones. This represents a substantial increase in the number of CA-MRSA clones and confirms their expansion in Kuwait hospitals. Secondly, whereas the proportion of ST80 isolates increased from 38.5% in 2001-2003 [17] to 51.1% in the present study, the proportion of ST30 isolates decreased from 30.8% in 2001-2003 to 22.2%. However, these changes were not statistically significant. The proportion of ST5 isolates has also increased from 7.7% in 2001-2003 to 14.1%. Thirdly, new clones, ST46, ST88, ST1, ST834 and ST950, appeared for the first time in Kuwait in this study. In contrast, strains belonging to ST361 and ST728 that were present in 2001-2003 were absent in this study highlighting the evolving nature of CA-MRSA isolates.

There was a high prevalence of resistance to cadmium acetate, kanamycin, fusidic acid, tetracycline, erythromycin, clindamycin and ciprofloxacin with 50.4% of the isolates being multiresistant to non-beta lactam antibiotics. Multiresistant-CA-MRSA isolates have also been reported among CA-MRSA clones isolated in Brazil [7], Japan [8] and USA [12].

We detected genes for PVL in 61.5% of the CA-MRSA isolates distributed among different sequence types as has also been reported in other countries [10,12,30,31]. We also detected agr type III genes in 67.4% of our CA-MRSA which is similar to results of studies in Belgium, France, Japan and Switzerland where the majority of their CA-MRSA isolates belong to agr type III [12,16] and possessed capsular polysaccharide type 8 [31]. However, whereas *sei *(63%) was the most common SE gene among our CA-MRSA isolates, *seg *was the most abundant SE gene in MRSA obtained in Ireland [30]. We observed the co presence of *seg *and *sei *in our isolates which has also been reported in CAMRSA studied in USA [32]. Furthermore, similar to the observation in CA-MRSA isolates from Ireland [30] and USA [32], none of our CA-MRSA isolates was positive for *see*. However, in contrast to the low prevalence of *tst *(6.7%) in this study, Rossney *et al., *[30] detected *tst *in 24% of CA-MRSA isolates in Ireland.

The ST80-SCC*mec*-IV clone has been the dominant CA-MRSA clone in European countries for some time [14,16]. With a prevalence of 51.1% of the CA-MRSA, the ST80 clone was the most common CA-MRSA clone in Kuwait hospitals. However, our ST80 isolates varied phenotypically. Although some were resistant to kanamycin, tetracycline and fusidic acid similar to the resistance pattern of the European ST80 clone [14,16], the results in Table 2 showed that some of our ST80 isolates had varied resistance patterns with some being susceptible to kanamycin, tetracycline and fusidic acid. They were also heterogeneous in their enterotoxin gene profiles. Remarkably, our ST80 isolates contained *sed, sei, seg, seb, seh *and *sea*, singly or in combinations. In contrast, studies from the Netherlands [16] detected only *seh *in their ST80 isolates. The detection of genes for different SEs in this study suggests that ST80 isolates may be acquiring genes for different SEs probably due to the acquisition of toxin-carrying bacteriophages [33]. In addition, 11 (16%) of the 69 ST80 isolates were negative for PVL genes. In a previous study, a small number (5 of 357) of ST80 isolates obtained from Algeria, Switzerland and France also lacked genes for PVL [34]. It is uncertain whether these isolates arose from PVL positive isolates due to the loss of the PVL phage or represents native ST80 backgrounds that had not previously acquired the PVL phage.

The dominance of the ST80 and ST30 CA-MRSA clones in Kuwait hospitals in 2001-2003 was attributed to their importation into the country by returning patients who sought medical care abroad, or expatriate staff from countries where these clones are prevalent [17]. Their presence in seven of the eight hospitals during the current study suggests that local transmission is also occurring. In addition, the diverse phenotypes observed with the ST80 isolates suggest that local variants of ST80 clones are emerging in Kuwait.

## Conclusion

This study has shown that the ST80-SCC*mec*-IV clone was the most common CA-MRSA clone in Kuwait hospitals in 2005-2006. However, other sequence types such as ST5 and ST6 also appeared. Most of the isolates were multi-resistant to antibacterial agents and harbored genes for different virulence factors. The successful expansion of CA-MRSA clones in Kuwait hospitals calls for novel approaches in infection control measures to control their spread.

## Competing interests

The authors declare that they have no competing interests.

## Authors' contributions

EEU designed the study, procured funding, guided the experimental work and wrote the first draft of the paper. ES contributed to the study design, performed experimental work and contributed to writing the manuscript. Both authors read and approved the final manuscript.
